# Management of Acne Vulgaris With Trifarotene

**DOI:** 10.1177/12034754231163542

**Published:** 2023-03-16

**Authors:** Jerry Tan, Rajeev Chavda, Hilary Baldwin, Brigitte Dreno

**Affiliations:** 1 70384 Windsor Clinical Research Inc, Ontario, Canada; 2 Department of Medicine, University of Western Ontario, Windsor Campus, Canada; 3 49549 Galderma, Switzerland; 4 Robert Wood Johnson Medical Center, New Brunswick, New Jersey, USA; 5 The Acne Treatment and Research Center, Brooklyn, New York, USA; 6 Dermato-cancerology Department, CHU Nantes, University of Nantes, France

**Keywords:** Acne vulgaris, trifarotene, topical retinoids, truncal acne, facial acne

## Abstract

Topical retinoids have an essential role in treatment of acne. Trifarotene, a topical retinoid selective for retinoic acid receptor (RAR) γ, is the most recent retinoid approved for treatment of acne. RAR-γ is the most common isoform of RARs in skin, and the strong selectivity of trifarotene for RAR-γ translates to efficacy in low concentration. Trifarotene, like other topical retinoids, acts by increasing keratinocyte differentiation and decreasing proliferation, which reduces hyperkeratinization. Retinoids have also been shown to inhibit inflammatory pathways via effects on leukocyte migration, toll-like receptors, and Activator Protein (AP)−1. Large-scale randomized, controlled clinical trials have demonstrated trifarotene to be safe, well tolerated, and efficacious in reducing both comedones and papules/pustules of acne. However, unlike all other retinoids, trifarotene is the first topical retinoid with rigorous clinical data on safety and efficacy in truncal acne. Data supporting use of trifarotene to manage acne are reviewed in this publication.

## Introduction

Acne vulgaris, a chronic inflammatory disease, is the 8th most prevalent disease worldwide and affects adolescents and adults across the demographic spectrum.^1^ Acne is also associated with long-lasting, sometimes permanent, sequelae including scarring and low self-esteem, particularly when acne affects highly visible regions such as the face.^2^ While it can affect any area rich with sebaceous glands - face, chest, shoulders and back—most research has focused on facial acne.^3^ However, the impact of truncal acne is becoming increasingly appreciated^4,5^ as approximately 50% to 66% of individuals with acne have trunk involvement.^3,6-9^ While almost half of all facial acne patients also have truncal acne, there has been a paucity of high quality studies on facial and truncal acne.^4^ In addition, recent studies have shown that facial and truncal acne together have a greater impact on quality of life compared to facial acne alone. Furthermore, the impact on quality of life worsens with increasing severity of truncal acne due to self-consciousness about appearance and reluctance to participate in activities where truncal acne would be revealed.^5^ Thus, truncal acne is an important and clinically relevant issue to address in the clinic.

Acne is a disease with a complex multifactorial pathogenesis, with four factors given most prominence: abnormal follicular keratinization, increased sebum production, colonization with *Cutibacterium acnes*, and immune system disturbances.^10^ Nutritional intake, namely a high glycemic index diet, and dairy intake may also affect acne.^11,12^ Genetics, the microbiome, and environmental influences additionally contribute.^13,14^

Clinically, active acne is associated with a morphologic spectrum of lesions, including papules, pustules, comedones, nodules and cysts. Acne lesions have traditionally been classified as “inflammatory” and “non-inflammatory;” however, inflammation exists at every stage of acne lesion development.^15,16^ Inflammatory mediators and receptors – including a range of cytokines, defensins, peptidases and other molecules—are activated during the initiation and propagation of acne.^15^ Uninvolved skin and early acne lesions show upregulation of E-selectin, vascular adhesion molecule-1, interleukin-1 (IL-1), and integrin.^15,17,18^ Further, IL-1 bioactivity has been shown in comedones, and upregulation of defensin-2 immunoreactivity and elevation of *T* cells and macrophages are found in uninvolved skin.^15,17-19^ Clinically, there has long been evidence that topical retinoids are effective in treating “noninflammatory” lesions, and it is known that retinoid therapy down-regulates toll-like receptor (TLR)−2 and IL-10 expression, thus acting in important pathways against inflammatory lesions.^20-23^ In addition, a recent transcriptomic study of changes in acne lesions that resolved spontaneously versus those that resolved during trifarotene treatment showed that trifarotene modulated a unique set of 67 genes that were not found in spontaneously resolving lesions.^24^ The genes affected by trifarotene were primarily involved in cellular migration, inflammation, and extracellular matrix organization and SPP1 +macrophages, a recently discovered proliferative macrophage found in fibrotic tissue.^24^

Topical retinoids are recommended as a foundation of acne treatment since they affect multiple aspects of acne pathophysiology.^2^ Since the US Food and Drug Administration (FDA) approval of tretinoin in 1971, retinoid molecules have undergone chemical modifications that have resulted in clinical improvements in efficacy and safety.^25^

Trifarotene 50 µg/g cream, a topical retinoid approved by the US FDA for once-daily treatment of acne, has been studied as treatment for both facial and truncal areas of involvement. A pharmacokinetic study (n = 19) showed that trifarotene has negligible systemic absorption after 29 days of topical application in doses ranging from 1.5 to 2 g per day to the face, chest, shoulders, and back. As a result, trifarotene did not reduce systemic exposure to oral contraceptives in 24 healthy volunteers participating in a drug-drug interaction study.^26^ Further, a human trial of 60 healthy adults found no effect on cardiac electrophysiology after 14 days of supra therapeutic dose trifarotene (12 g of trifarotene 100 µg/g).^22^ Trifarotene is a selective agonist of retinoic acid receptor (RAR) γ, the most common RAR found in the skin.^27^

This publication discusses clinical data supporting use of trifarotene, a novel topical retinoid, in treatment of acne.

## Clinical Data Supporting Use of Trifarotene in Acne

Trifarotene was evaluated in a clinical development program that included two large-scale phase 3 studies and a long-term safety study, among others (Table 1). Unique among modern acne products, the efficacy of trifarotene was rigorously studied on the trunk in addition to the face.^28^ Recently, recommendations were made about truncal acne by the Personalising Acne: Consensus of Experts (PACE) panel. These included encouraging healthcare professionals to prompt discussion about truncal acne, to grade truncal acne severity separately from facial acne, to individualize treatment based on impact of acne in different regions, and to consider risk for scarring.^29^

**Table 1:** Key Results for Pivotal Trifarotene Clinical Trials in Acne.

Phase 3 trials: PERFECT 1 and PERFECT 2 (12 weeks) All endpoints significantlyin favor of trifarotene vs vehicle (*P* < .001)				
	PERFECT 1 (NCT02566369)		PERFECT 2 (NCT02566788)	
	Trifarotene (n = 612)	Vehicle (n = 596)	Trifarotene (n = 602)	Vehicle (n = 610)
IGA success^a^ (face) PGA success^b^ (trunk)	29.4% 35.7%	19.5% 25.0%	42.3% 42.6%	25.7% 29.9%
**SATISFY Long-term study (52 weeks**, n = 453) (NCT02189629)				
		Week 12	Week 26	Week 52
IGA success (face) PGA success (trunk) Pt reported marked/complete improvement (face)		26.6% 38.6% 41.4%	50.1% 58.4% 54,8%	65.1% 66.9% 66.6%

Abbreviations: IGA, Investigator’s global assessment; PGA, Physician’s global assessment (trunk).

^a^IGA rating of clear/almost clear (0/1) plus improvement of at least 2 grades

^b^PGA rating of clear/almost clear (0/1) plus ≥grade improvement

### Pivotal Studies

Trifarotene was studied in two identical, 12 week phase 3 studies in patients with moderate facial and truncal acne (PERFECT 1 and 2).^28^ Acne patients included had moderate facial acne defined as an investigator global assessment (IGA) score of 3 on the face, with at least 20 inflammatory lesions and ≥25 non-inflammatory lesions; they also had moderate truncal acne with a physician global assessment (PGA) score of 3 at screening with ≥20 inflammatory lesions and 20-100 non-inflammatory lesions.^28^ Patients with severe acne, acne cysts, or more than one nodule on face or trunk were excluded. A total of 2,420 patients aged 9 years or older (1,214 treated with trifarotene and 1,206 treated with vehicle) were randomized to once-daily trifarotene 50 µg/g or vehicle cream.^28^ Demographic and acne characteristics were similar between treatment groups.^28^

Efficacy results on global assessments at week 12 are shown in Table 1; facial acne outcomes (primary) and truncal outcomes (secondary) efficacy assessments in both studies were statistically significantly (*P* < .001) in favor of trifarotene compared to vehicle.^28^ Statistical separation between groups in achieving facial global success (clear or almost clear) were reported at week 4 in PERFECT 1 and week 8 in PERFECT 2. Compared to baseline, mean reduction in facial lesion counts were higher in the trifarotene group (not shown in Table 1), with statistically significant differences by week 1 (*P* < .001). Truncal acne also responded well to trifarotene therapy. Separation between groups in reduction of truncal lesions was apparent by week 4 in PERFECT 1 and as early as week 2 in PERFECT 2.^28^

**Figure 1:**
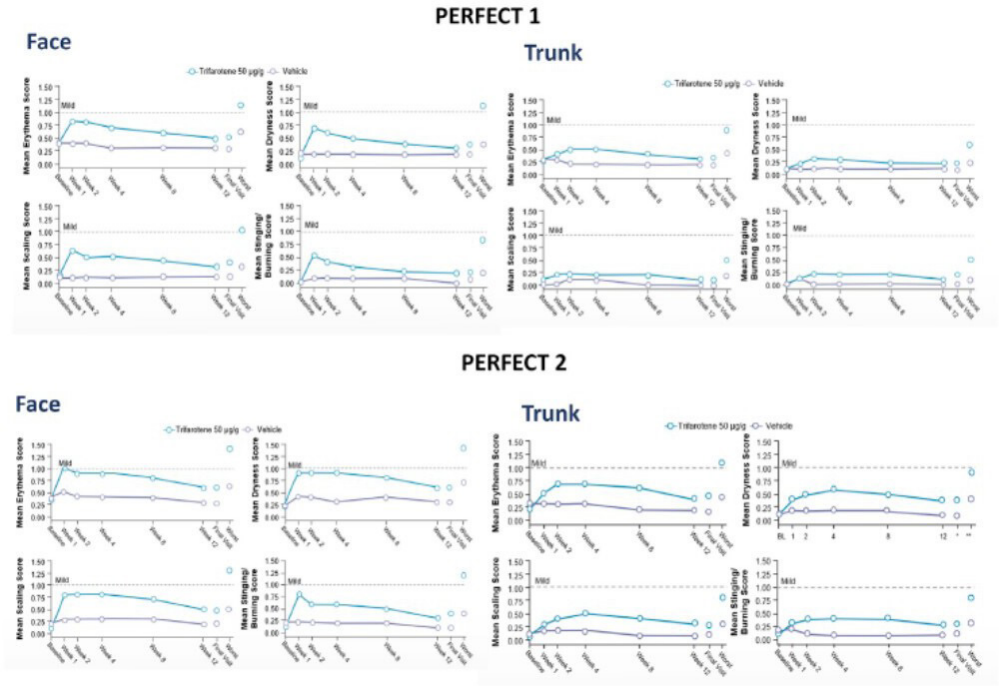
Acne vulgaris. Local tolerability in phase 3 clinical trials of trifarotene in acne. Severity scale score indicates: 0 (none), 1 (mild), 2 (moderate), and 3 (severe). From Tan et al.

Adverse events were mild to moderate, and, when present, diminished after the first weeks of use.^28^ Most commonly reported were sunburn (2.6%, 0.5% vehicle), application site irritation (7.5%, 0.3%) and application site pruritus (2.4%, 0.8%). Local tolerability was consistent with transient topical retinoid dermatitis (Figure 1). Overall tolerability was better on the trunk than face. Irritation scores reached maximum severity by week 1 for the face, and by week 2-4 on the trunk, then diminished. Overall, severe cutaneous adverse events occurred in 9 patients (including skin irritation, sunburn, allergic dermatitis, application site pain/erosion/irritation) but no serious adverse events were reported. Trifarotene therapy was discontinued because of adverse events by 1.2% (PERFECT 2) and 1.9% (PERFECT 1) of patients. Thus, trifarotene had a safety and tolerability profile consistent with topical retinoids, and most adverse events were cutaneous irritation in the first few weeks of treatment and improvement with continued therapy.^28^

The long term trial was a 52 week multicenter, open-label study (n = 453) in moderate facial and truncal acne treated with trifarotene cream.^30^ Global success (rating of 0/1 or clear/almost clear) was attained in 26.6% at the face and 38.6% at the trunk by week 12.^30^ Continued improvement was reported over the duration of the study. At week 52, 65.1% achieved facial global success and 66.9% truncal global success.^30^ In addition, 57.9% of patients had both facial and truncal clearance of acne lesions.^30^ As in the other phase 3 studies, the safety and tolerability of trifarotene was good; adverse events were reported in 12.6% of patients.^30^ Most adverse event involved cutaneous tolerability and resolved with ongoing treatment.^30^ There was a completion rate of 76.5% in this 52 week study, which is notable, given the majority of patients were younger than 18 years.^30^ Further, trifarotene treatment was associated with marked improvement in quality of life as shown in DLQI scores (with 53.8% of patients reporting no effect of acne [score 0/1] at week 52 versus 22.6% at baseline).^30^ This is consistent with Newton et al, who reported successful treatment of acne led to improved quality of life and “disability caused by acne can be largely reversed by effective treatment”^31^

### Combination Therapy: Trifarotene Plus Doxycycline in Severe Acne

**Figure 2:**
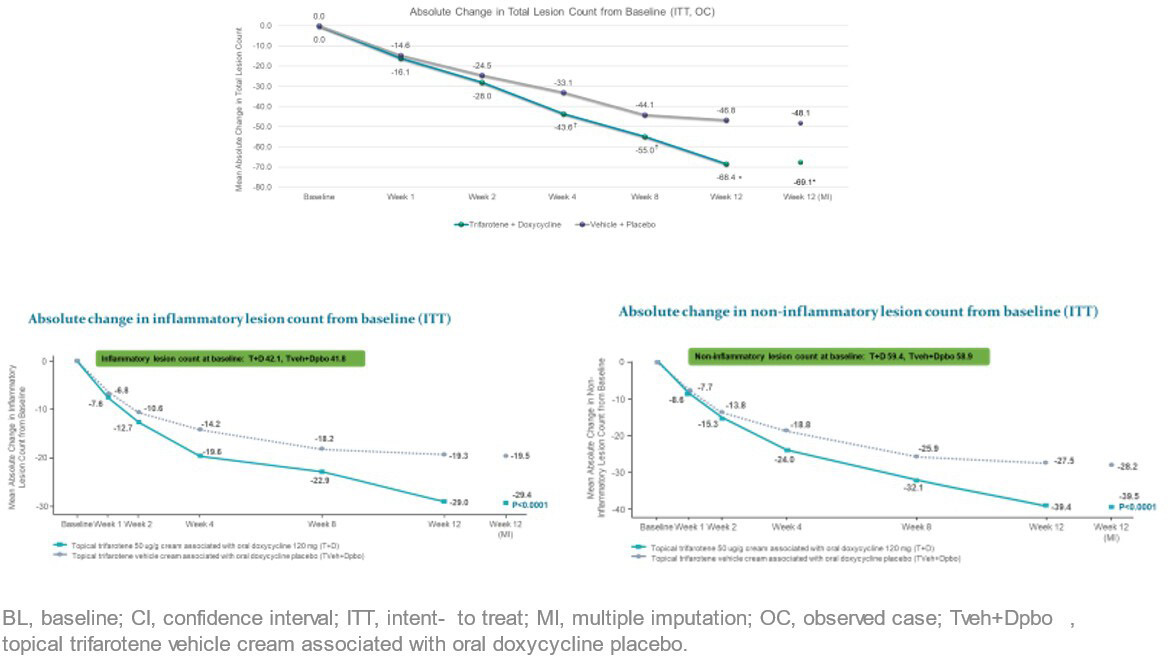
Acne vulgaris. Reduction in acne lesion counts (absolute numbers) in patients treated with trifarotene plus doxycycline vs vehicle plus placebo. From Del Rosso et al.

**Figure 3:**
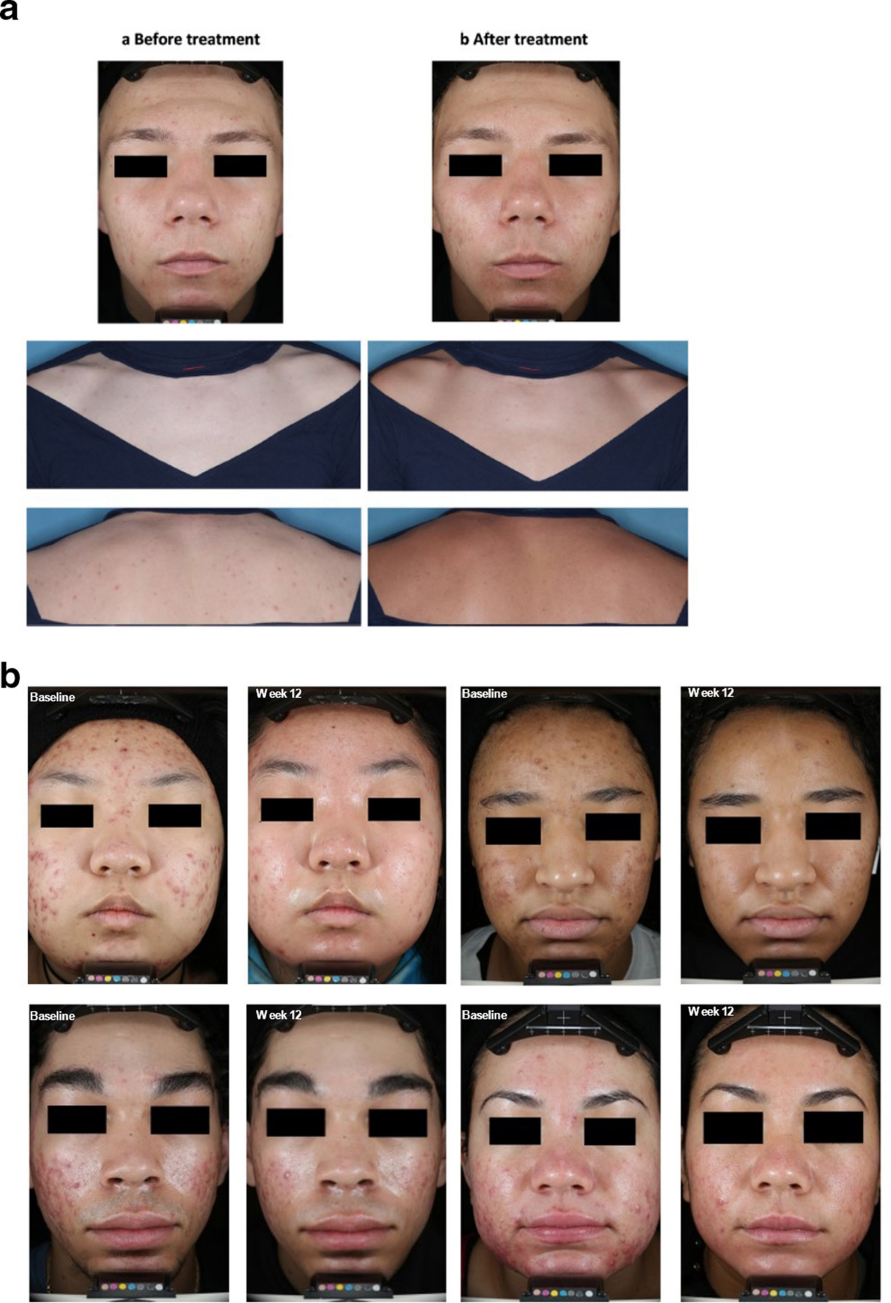
Acne vulgaris. Patient photos (a) trifarotene monotherapy (From Johnson et al) and (b) trifarotene plus doxycycline in severe acne. From Del Rosso et al.

Efficacious management of moderate to severe acne is pivotal to reducing long-term consequences. Layton et al. recently published an expert panel’s recommendations to reduce burden of acne sequelae, including erythema, hyperpigmentation, and scarring.^32^ These researchers focused on the need to discuss acne sequelae with patients, and recommended healthcare professionals assess risk for scarring, erythema, and hyperpigmentation at diagnosis, with discussions of family history of acne and its consequences as appropriate. They stressed the need to “relieve the patient burden of acne sequelae through improved risk factor recognition, early discussion, and prevention at all stages of the patient journey.”^32^ The combination of trifarotene cream plus oral doxycycline 120 mg has been studied in severe acne.^33^ Patients ≥ 12 years old with severe facial acne defined as ≥20 inflammatory lesions, 30-120 non-inflammatory lesions, and up to 4 nodules were randomized 2:1 to receive either trifarotene cream plus doxycycline 120 mg (n = 133, *T* + D) or trifarotene vehicle and doxycycline placebo (n = 69, V + *P*) for 12 weeks. Absolute total lesion counts changed by −69.1 in the *T* + D group and −48.1 in the V + *P* group (Figure 2, *P* < .0001). Superior reductions in both inflammatory and non-inflammatory lesions were also reported with the *T* + D combination. Success (IGA 0/1) was reported in 31.7% of patients in the *T* + D group vs 15.8 of those in the V + *P* group (*P* = .01). Figure 3 presents patients before and after treatment either with trifarotene (a) or the combination of trifarotene and doxycycline (b). Safety and tolerability were similar between treatment groups, with adverse events occurring in 13.5% of patients treated with *T* + D and 15.9% of those treated with V + P.^33^

## Conclusions

Trifarotene is a recently approved retinoid selectively targeting RAR-γ receptors of the skin.^24^ It has been shown to be efficacious in facial and truncal acne and safe for long-term maintenance with favorable efficacy and tolerability.^34^ As monotherapy for acne, continuous improvement over time was reported in the long-term safety study according to both investigators and patients. Trifarotene was recently shown to be efficacious in combination with oral doxycycline, extending its use to patients with severe acne. The trifarotene formulation is reported by patients to be easily spreadable, has an elegant texture suited for face and trunk application, and is associated with low systemic exposure.^27^ Trifarotene studies showed that some patients treated with trifarotene vehicle alone also had improvements; we feel this is common in acne trials and likely at least partly due to the controlled conditions of a clinical trial and optimization of skin regimens. Overall, trifarotene is an important treatment option for acne vulgaris in patients 9 years or older, and is suitable for monotherapy in moderate acne on both the face and trunk, and in combination with oral doxycycline may be considered for severe acne.

## Supplemental Material

Figure S1 - Supplemental material for Management of Acne Vulgaris With Trifarotene Click here for additional data file. Supplemental material, Figure S1, for Management of Acne Vulgaris With Trifarotene by Jerry Tan, Rajeev Chavda, Hilary Baldwin and Brigitte Dreno in Journal of Cutaneous Medicine and Surgery

Figure S2 - Supplemental material for Management of Acne Vulgaris With Trifarotene Click here for additional data file. Supplemental material, Figure S2, for Management of Acne Vulgaris With Trifarotene by Jerry Tan, Rajeev Chavda, Hilary Baldwin and Brigitte Dreno in Journal of Cutaneous Medicine and Surgery
