# Supplementary material for: Management of Acne Vulgaris With Trifarotene
Source: J Cutan Med Surg. 2023 Mar 16;27(4):368–74. doi: 10.1177/12034754231163542 (PMC10486177; doi:10.1177/12034754231163542)
Supplement: Figure S1 - Supplemental material for Management of Acne Vulgaris With Trifarotene [file sj-pdf-1-cms-10.1177_12034754231163542.pdf]

RightsLink

Home | Help | Live Chat | Valerie Sanders

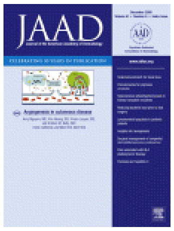

Randomized phase 3 evaluation of trifarotene 50 µg/g cream treatment of moderate facial and truncal acne

Author:
Jerry Tan,Diane Thiboutot,Georg Popp,Melinda Gooderham,Charles Lynde,James Del Rosso,Jonathan Weiss,Ulrike Blume-Peytavi,Jolanta Weglovska,Sandra Johnson,Lawrence Parish,Dagmara Witkowska,Nestor Sanchez Colon,Alessandra Alió Saenz,Faiz Ahmad et al.

Publication: Journal of the American Academy of Dermatology

Publisher: Elsevier

Date: June 2019

© 2019 by the American Academy of Dermatology, Inc.

Order Completed

Thank you for your order.

This Agreement between Valerie J Sanders ("You") and Elsevier ("Elsevier") consists of your license details and the terms and conditions provided by Elsevier and Copyright Clearance Center.

Your confirmation email will contain your order number for future reference.

Printable Details

**License Number**	5490871290299
**License date**	Feb 16, 2023

Licensed Content

Licensed Content Publisher	Elsevier
Licensed Content Publication	Journal of the American Academy of Dermatology
Licensed Content Title	Randomized phase 3 evaluation of trifarotene 50 μg/g cream treatment of moderate facial and truncal acne
Licensed Content Author	Jerry Tan,Diane Thiboutot,Georg Popp,Melinda Gooderham,Charles Lynde,James Del Rosso,Jonathan Weiss,Ulrike Blume-Peytavi,Jolanta Weglovska,Sandra Johnson,Lawrence Parish,Dagmara Witkowska,Nestor Sanchez Colon,Alessandra Alió Saenz,Faiz Ahmad et al.
Licensed Content Date	Jun 1, 2019
Licensed Content Volume	80
Licensed Content Issue	6
Licensed Content Pages	9

Order Details

Type of Use	reuse in a journal/magazine
Requestor type	academic/educational institute
Portion	figures/tables/illustrations
Number of figures/tables/illustrations	1
Format	both print and electronic
Are you the author of this Elsevier article?	Yes
Will you be translating?	No

About Your Work

Title of new article	Selection of nemolizumab clinical dose for atopic dermatitis
Lead author	Nathalie Wagner
Title of targeted journal	Dermatology and Therapy
Publisher	SanovaWorks
Expected publication date	Apr 2023

Additional Data

Portions	Figure 3

Requestor Location

Requestor Location	Valerie J Sanders<br>30 Forest St<br><br>MONTCLAIR, NJ 07042<br>United States<br>Attn: Valerie J Sanders

Tax Details

Publisher Tax ID	98-0397604

Price

Total	0.00 USD

Total: 0.00 USD

CLOSE WINDOW ORDER MORE

© 2023 Copyright - All Rights Reserved | Copyright Clearance Center, Inc. | Privacy statement | Data Security and Privacy | For California Residents | Terms and Conditions

Comments? We would like to hear from you. E-mail us at customercare@copyright.com
