# Supplementary material for: Management of Acne Vulgaris With Trifarotene
Source: J Cutan Med Surg. 2023 Mar 16;27(4):368–74. doi: 10.1177/12034754231163542 (PMC10486177; doi:10.1177/12034754231163542)
Supplement: Figure S2 - Supplemental material for Management of Acne Vulgaris With Trifarotene [file sj-pdf-2-cms-10.1177_12034754231163542.pdf]

CCC Marketplace

Order Confirmation

Thank you, your order has been placed. An email confirmation has been sent to you. Your order license details and printable licenses will be available within 24 hours. Please access Manage Account for final order details.

This is not an invoice. Please go to manage account to access your order history and invoices.

CUSTOMER INFORMATION

Payment by credit card: Your order will be finalized and your card will be charged within 24 hours.

Billing Address

Valerie Sanders
491 S Woodstock Dr
PUEBLO, CO 81007
United States

+1 (850) 258-0013
vjsnders@aol.com

Customer Location

Valerie Sanders
6818 Forsythe Dr
Panama City, FL 32404
United States

PO Number (optional)

N/A

Payment options

CC ending in 9548

PENDING ORDER CONFIRMATION

Confirmation Number: Pending

Order Date: 20-Feb-2023

1. The Journal of clinical and aesthetic dermatology

53.50 USD

Article: A Randomized, Controlled Trial of Trifarotene Plus Doxycycline for Severe Acne Vulgaris.

**Order License ID**	Pending	**Publisher**	Matrix Medical Communications
**ISSN**	1941-2789		
**Type of Use**	Republish in a journal/magazine	**Portion**	Image/photo/illustration

LICENSED CONTENT

**Publication Title**	The Journal of clinical and aesthetic dermatology
**Article Title**	A Randomized, Controlled Trial of Trifarotene Plus Doxycycline for Severe Acne Vulgaris.
**Date**	01/01/2008
**Language**	English
**Country**	United States of America
**Rightsholder**	Matrix Medical Communications
**Publication Type**	Journal
**Start Page**	E53
**End Page**	E59
**Issue**	7
**Volume**	15

REQUEST DETAILS

**Portion Type**	Image/photo/illustration
**Number of Images / Photos / Illustrations**	2
**Format (select all that apply)**	Print, Electronic
**Who Will Republish the Content?**	Publisher, for profit
**Duration of Use**	Life of current edition
**Lifetime Unit Quantity**	Up to 999
**Rights Requested**	Main product
**Distribution**	Worldwide
**Translation**	Original language of publication
**Copies for the Disabled?**	No
**Minor Editing Privileges?**	No
**Incidental Promotional Use?**	No
**Currency**	USD

NEW WORK DETAILS

**Title**	Management of acne vulgaris with trifarotene
**Author**	Jerry Tan, MD
**Publication**	Journal of Clinical Medicine and Surgery
**Publisher**	Sage
**Publisher Imprint**	N/A
**Expected Publication Date**	2023-03-06
**Expected Size of the New Work (number of pages)**	5
**Standard Identifier**	N/A

ADDITIONAL DETAILS

**Order Reference Number**	N/A
**The Requesting Person/Organization to Appear on the License**	Jerry Tan, MD

REQUESTED CONTENT DETAILS

**Title, Description or Numeric Reference of the Portion(s)**	Figures 1 and 2
**Title of the Article/Chapter the Portion Is From**	A Randomized, Controlled Trial of Trifarotene Plus Doxycycline for Severe Acne Vulgaris.

**Editor of Portion(s)**	Del Rosso, James Q; Johnson, Sandra Marchese; Schlesinger, Todd; Green, Lawrence; Sanchez, Nestor; Lain, Edward; Draelos, Zoe; York, Jean-Philippe; Chavda, Rajeev	**Author of Portion(s)**	Del Rosso, James Q; Johnson, Sandra Marchese; Schlesinger, Todd; Green, Lawrence; Sanchez, Nestor; Lain, Edward; Draelos, Zoe; York, Jean-Philippe; Chavda, Rajeev
**Volume of Serial or Monograph**	15	**Issue, if Republishing an Article From a Serial**	7
**Page or Page Range of Portion**	E53-E59	**Publication Date of Portion**	2022-06-30

Total Items: 1

Total Due: 53.50 USD

Accepted: Marketplace Permissions General Terms and Conditions and any applicable Publisher Terms and Conditions
